# Response to: Understanding the null hypothesis (H0) in non-inferiority trials

**DOI:** 10.1186/s13054-017-1790-2

**Published:** 2017-08-04

**Authors:** Xiang Zhou, Dawei Liu, Longxiang Su

**Affiliations:** Department of Critical Care Medicine, Peking Union Medical College Hospital, Peking Union Medical College & Chinese Academy of Medical Sciences, Beijing, 100730 China

We thank Dr. Mallat for their interest in our study of stepwise lactate kinetics-oriented hemodynamic therapy and for putting forward a statistical problem [1]. Central venous oxygen saturation (ScvO2)-targeted therapy was recommended by the Surviving Sepsis Campaign (SCC) guidelines in 2012 [2] and we initially envisaged that the stepwise lactate kinetics strategy was, at least, not inferior to the ScvO2 target strategy. Therefore, the design of non-inferiority analysis was adopted at the beginning of the trial.

We rechecked and recalculated the data by non-inferiority and superiority test. The lactate kinetics group mortality rate (P1) was 18.3% and the Scvo2 group mortality rate (P2) was 27.9%. The standard error for the mortality difference between the two groups was 0.0441, and thus the mortality difference was –0.0944 (95% confidence interval –0.1809 to –0.0080). The upper limit of the interval is less than 0, and thus the superiority conclusion is established. At this point, regardless of the non-inferior value (0.15 or 0.10), non-inferior conclusions must be established.

Additionally, the non-inferiority threshold of 10% is indeed more reasonable according to a previous study [3].Therefore, regardless of whether the non-inferiority threshold is set at 10% or 15%, no effect on the final conclusion was seen in this study.

Last, but not the least, the latest sepsis guidelines released by the SCC in 2016 have weakened early goal-directed therapy (EGDT) and highlighted the importance of normalization of lactate [4], which also supports the conclusion we draw that stepwise lactate kinetics-oriented hemodynamic therapy can reduce mortality in patients with sepsis-associated hyperlactatemia compared with ScvO2-oriented therapy.

## Abbreviations

EGDT: Early goal-directed therapy
SCC: Surviving Sepsis Campaign
ScvO2: Central venous oxygen saturation
